# Sex Variability in Pediatric Leukemia Survival: Large Cohort Evidence

**DOI:** 10.5402/2012/439070

**Published:** 2012-04-03

**Authors:** L. Holmes, J. Hossain, M. desVignes-Kendrick, F. Opara

**Affiliations:** ^1^American Health Research Institute, Heights Medical Tower, Houston, TX 77008, USA; ^2^Nemours Center for Childhood Cancer Research, Wilmington, DE 19803, USA; ^3^Orthopedic Department, Epidemiology & Biostatistics Section, A.I.duPont Hospital for Children, Wilmington, DE 19803, USA; ^4^University of Delaware, Newark, DE 19716, USA; ^5^Biomedical Research Department, A.I.duPont Hospital for Children, Wilmington, DE 19803, USA; ^6^Texas A & M University, Houston, TX 77030, USA

## Abstract

*Purpose*. Sex disparities in pediatric leukemia have been previously reported, and male children continue to present with poorer survival. However, the observed disparities are not fully understood. This current study sought to examine disparities in survival by the sex, and to determine if tumor prognostic factors impact on these disparities. *Patients and Methods*. We used the Surveillance Epidemiology and End Results dataset of pediatric leukemia patients (ages 0–19 years) diagnosed in the United States from 1973 to 2006. There were 15,215 patients of whom 8,622 (65.7%) were boys and 6,593 (43.3%) were girls. The Kaplan-Meier survival estimates, log rank test, and Cox proportional hazard methods were used to assess the data. *Results*. The overall (both sexes) five-year survival rate was 67.9%. Girls had a survival rate of 70.1%, while the rate was 66.3% in boys. Girls had a significant 14% decreased risk of dying relative to boys, hazard ratio (HR) = 0.86, 99% CI = 0.80–0.93. There were significant differences between boys and girls with respect to tumor cell type, race, age at diagnosis, year of diagnosis, and number of primaries, *P* < 0.001. After controlling for these factors, the sex differences in survival persisted, with girls still less likely to die from leukemia compared to boys, adjusted HR (AHR) = 0.85, 99% CI = 0.72–1.00, *P* < 0.01. *Conclusion*. In a large population-based pediatric leukemia study, boys continued to show poorer survival. These disparities were not completely explained by treatment received, tumor prognostic or socio-demographic factors.

## 1. Introduction

Leukemia remains the most frequently diagnosed childhood malignancy in the United States, and while overall survival has increased, the incidence remains to plateau [1–3]. The improvement in survival may be due to both the early stage at diagnosis and advances in therapy [3]. However, despite overall improvements in survival, one segment of children still experience poorer survival, mainly male children. In addition, survival is poorest among black children. This survival disadvantage of both male sex and black ethnicity/race may be explained by the type of treatment received, unfavorable biological features, stage of tumor at diagnosis, age at diagnosis, comorbidity, and availability and timely access to treatment [1–14].

Even in the era of modern advances in treatment and protocols (since the early 1960s), male children diagnosed with and treated for leukemia continue to show poorer prognosis [3–17]. The observed sex differentiation in pediatric leukemia survival may be related to tumor prognostic factors such as the cell type—T cells versus B cells/B cell precursor. There are no studies to our knowledge that are both population-based and contain a large sample that have assessed sex as an independent survival factor. The Surveillance Epidemiology and End Results (SEER) is an example of a large database that allows for the assessment of the effect of sex on cancer survival. Because of the limited focus in the literature on the persistent sex variance in pediatric leukemia survival, we hypothesized that sex differences continue to impact survival, and that this variation may be explained by biologic features. We sought in this study to assess the effect of sex on pediatric leukemia survival using over thirty years of data from SEER. Further, we sought to examine tumor prognostic factors that may possibly remove the significant difference in mortality.

## 2. Patients and Methods

The SEER datasets from the 17 registries were used to examine the impact of sex on survival of patients diagnosed with leukemia and treated for the disease. While leukemia is not a homogenous cancer, we wanted to assess the effect of sex on survival from all leukemias. This approach was taken to ensure a large sample for this study. From 1973 to 2006, there were 15,321 children diagnosed with leukemia (clinical subtypes combined).

### 2.1. Data Source

We used the SEER database which includes information from 17 registries. This database is estimated to represent 26% of the US population. SEER has information on tumor histology, number of primary tumors, radiation therapy, surgery, survival status, and survival time but includes no information on chemotherapy. Demographic information is available on age at tumor diagnosis, year of diagnosis, sex, and race. The SEER dataset is known to be reliable and valid for the conduct of population-based studies involving cancer in the United States [18–20].

### 2.2. Diagnosis

Leukemia was ascertained using the International Classification of Diseases and Related Health Problems, 10th revision (ICD-10). The clinical subtypes were also ascertained using the same classification code.

### 2.3. Study Variables

#### 2.3.1. Sex

Sex in the SEER dataset and for the purpose of this study is a biological construct and refers to the classification of living things, generally as male or female according to their reproductive organs and functions assigned by their chromosomes. We treated the male as the reference group with this dichotomous nominal variable.

#### 2.3.2. Race

The SEER dataset collects information on race as (a) white, (b) black, (c) others, and (d) unknown. Because of the difficulties in explaining others and unknown, we did not stress the latter two groups in the interpretation of the results in this study.

#### 2.3.3. Age at Diagnosis

We examined the age at diagnosis of patients and extracted data from all patients 0 to 19 years of age at the time of diagnosis. Age is recorded in category namely: (a) <1 year, (b) 1–4, (c) 5–9, (d) 10–14, and (d) 15–19 years. These age categories represent pediatric malignancies groupings used in most studies and were adopted for the purpose of this paper.

#### 2.3.4. Year of Diagnosis

This study covered 34 years of data collected by several SEER registries (9–17 over time). The details of the SEER registries are available elsewhere [21]. We examined every year for which leukemia was diagnosed as well as mortality status of the patients. To provide some insight into the survival of these patients by group of years of diagnosis, we created five-year interval categories (except for the last group, 2003–2006) of the year of diagnosis (1973–1977, 1978–1982, 1983–1987, 1988–1992, 1993–1997, 1998–2002, and 2003–2006). These categories simulate the five-year survival periods commonly used in assessing the clinical benefits of cancer therapeutics. For the purpose of the analysis, we treated year of diagnosis as a single and categorical year in order to examine the patterns of survival. Because the year of diagnosis may influence the treatment pattern and hence prognosis, as well as reflect time dependency (time-dependent variable), we used this variable in the stratified analysis (Stratified Cox).

#### 2.3.5. Number of Primaries

The SEER dataset collects information on the number of primary tumors as (a) 1, (b) 2, and (c) 3 primaries. For the purpose of this study, we treated this variable as binary by creating two groups of primaries, namely, (a) one primary and (b) two or more primaries, with one primary set as the reference group in the analysis.

### 2.4. Tumor Cell Types

The cell type of leukemia is available in the SEER dataset. We extracted information on this variable and used two distinct cell types, namely, T cell and B cell/B precursor.

This variable was treated as binary, with the T cell as the reference group.

### 2.5. Radiation Therapy

The SEER dataset lists information of radiation therapy in a nominal pattern. Radiation is grouped into (a) beam radiation (b) combination, meaning beam radiation with implant or isotopes, (c) radiation NOS, method, or source not specified, (d) recommended, but unknown if administered, (e) refused, and (f) unknown. Detailed information on the radiation therapy regimen such as dosage is not available. This variable was dichotomized into (a) radiation = yes and (b) no radiation = no.

### 2.6. Survival Time and Status

The survival time is listed as months from the time of diagnosis to the time death from any cause. In the dataset, those who did not experience the event (death) during the followup time were censored. The followup time is listed as the duration from time of diagnosis to death from any cause or last day of the availability of survival information in the SEER registry. Therefore, the followup time varies, with the earlier diagnosed patients having longer followup times compared to those diagnosed later. For example, a patient who was diagnosed with leukemia in January 1973 and was still alive in 2006 has a maximum followup time of 408 months (1973–2006: 408 months). The survival status was measured on a binary scale, with 0 (zero) for censored and 1 (one) for the event or failure.

### 2.7. Statistical Analyses

We performed a preanalysis screening by examining data for missing values. To examine the association between sex and the study variables, we used a chi-square statistic. And to assess the effect of individual covariates on survival, we utilized the univariable Cox proportional hazard method and obtained the hazard ratio (relative risk of dying that reflects the magnitude of the association between covariate and survival) as a point estimate and 99% confidence interval (CI) as well as the *P* value for statistical stability of our point estimate. Because there are many factors that influence survival of a cohort of cancer patients treated for the disease, we examined the effect of sex in combination with other confounding factors using the multivariable Cox proportional hazard method. In this regard, we performed two adjusted models, one without and the other with tumor cell types, and provided two results in this study. Further, we performed the same analyses stratified by the single year of diagnosis which allowed us to compare hazard of a given year with the corresponding year's baseline hazard. Finally, we used the log rank test to examine the equality of survival by sex. Also, we graphically illustrated survival estimates in the overall group as well as by sex using the Kaplan-Meier survival curves and survival proportion curve from the life table. The significance level was 0.01, and all tests were two-tailed. The Statistical Package for Social Sciences (SPSS), version 17.0, SPSS Inc., Chicago, Illinois and STATA (StataCorp) version 11.0, College station, TX were used to perform the analysis.

## 3. Results

Of the 15,215 children diagnosed with leukemia from 1973 to 2006 in the SEER database, 8622 (56.7%) were boys and 6593 (43.3%) were girls.  Table 1(a) presents demographic and tumor factors by sex. There were no sex differences by race, year of diagnosis, and number of primaries, *P* > 0.01. However, there were significant differences in age at diagnosis ( = *χ* ^2^ 49.8(4), *P* < 0.0001) and mortality status (*χ* ^2^ = 23.6(1), *P* < 0.0001) comparing boys with girls. Similarly, boys and girls did differ significantly with respect to the radiation therapy received, (*χ* ^2^ = 23.2(1), *P* < 0.0001). Further, there was a statistically significant difference, comparing boys with girls with respect to the cell types of leukemia (T cell versus B cell/B precursors), (*χ* ^2^ = 68.7(1), *P* < 0.0001). The 5-year overall survival rate was 67.9%, while the 30-year survival rate was 55.6%. Girls showed a better 5-year survival rate of 70.1%, while their 30-year survival was 58.0%. Survival was poorer among boys, with a 5-year survival rate of 66.3%, while their 30-year survival rate was 53.9% (Table 1(b)).

Table 2 shows the factors associated with mortality in our cohort. In this univariable and crude Cox proportional hazard model, there was a statistically significant difference in the mortality outcome in children with leukemia, comparing the two sexes. Relative to boys, girls were 14% less likely to die from leukemia following the diagnosis and treatment, hazard ratio (HR = 0.86), 99% Confidence Interval (CI), 0.82–0.92. Black children did differ from whites with respect to mortality and were 54% more likely to experience mortality, HR = 1.54, 99% CI, 1.40–1.69. Radiation per se did not significantly improve survival, but rather survival was worsened when considering this as a monotherapy, HR = 0.84, 99% CI = 0.77–0.92, *P* < .0001, implying that children with leukemia who did not receive radiation but received other treatments were 16% less likely to have failure or experience the event.

Survival also varied significantly by age at diagnosis using the standard childhood cancer age grouping. Compared to children who were less than one year of age, children 1 to 4 years were 72% less likely to die from leukemia, HR = 0.28, 99% CI, 0.23–0.29. Children 5 to 9 years were 67% less likely to die (HR = 0.33, 99% CI 0.30–0.37). Children ages 10–14 were 44% less likely to die compared to the children <1 year of age (HR = 0.56, 99% CI 0.50–0.63). Also, using the dichotomous age grouping in order to examine the possible effect of estrogen or testosterone, there was a significant difference in mortality by age at diagnosis comparing children <1.0–9 years to children 10–19 years, HR = 2.44, *P* < 0.0001. Mortality experience did differ significantly by year of diagnosis, with mortality reduction observed during the later years of diagnosis relative to former. Using the 5-year interval with 1973–1977 as the reference year, a significant decrease in mortality trend was observed (*P* value for trend, <0.001). There was a significant 75% increase in mortality, comparing two or more primaries to one, and children with two or more primaries were 75% more likely to die, HR = 1.75, 99% CI = 1.47–2.09.

In assessing the confounding effect of race, age at diagnosis, year of diagnosis, radiation therapy, and the number of primaries, the association between sex and leukemia mortality among children persisted, adjusted hazard ratio (AHR) = 0.88, 99% CI = 0.83–0.93. Similarly, after further adjustment including the cell type of leukemia (T cell versus B cell/B precursors), the significant relationship between sex and pediatric leukemia survival further persisted, AHR = 0.85, 99% CI 0.72–1.00, *P* = 0.01. Whereas the radiation treated as monotherapy did not favor survival, after adjustment for other covariates with prognostic potentials in pediatric leukemia, there was a significant 21% increased risk of dying among those who did not receive radiation therapy, AHR = 1.21, 99% CI 1.10–1.23, *P* < 0.0001 (Table 3). Since the year of diagnosis is relevant to survival due to its prognostic effect (improvement in treatment protocol overtime), which may serve as a time-dependent covariate, and adjustment by five-year interval has a likelihood of introducing residual confounding into the results, we stratified the analysis by the year of diagnosis. This approach was taken in order to compare the hazard of a given year to the baseline hazard of the same year, thus removing the effect of the year of diagnosis on the effect of sex on the survival. With this stratification, survival difference by sex persisted without leukemia cell type (AHR = 0.86, 99% CI = 0.81–0.94, *P* = 0.018) and with leukemia cell types (AHR = 0.85, 99% CI = 0.72–1.01, *P* = 0.018).

**Figure 1:**
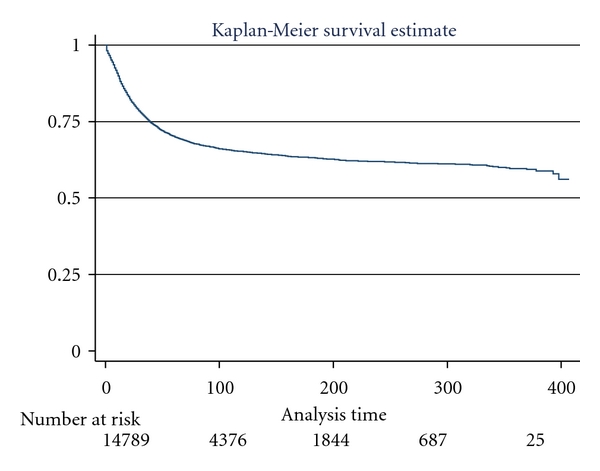
Kaplan-Meier survival curve for all pediatric patients with leukemia, SEER dataset 1973–2006.

**Figure 2:**
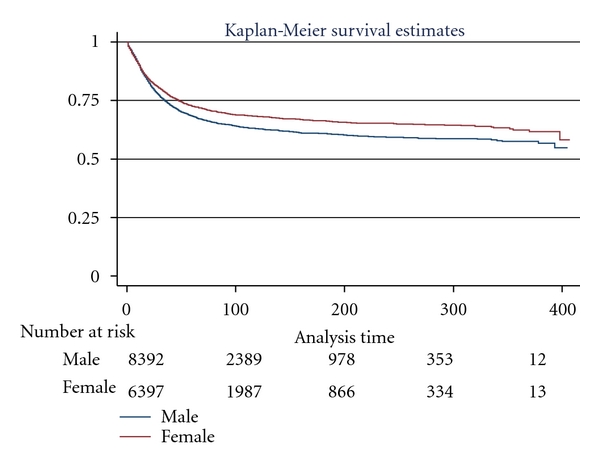
Kaplan-Meier survival curve illustrating distinct survival of pediatric patients with leukemia by sex, (log rank *P* < 0.0001).

**Figure 3:**
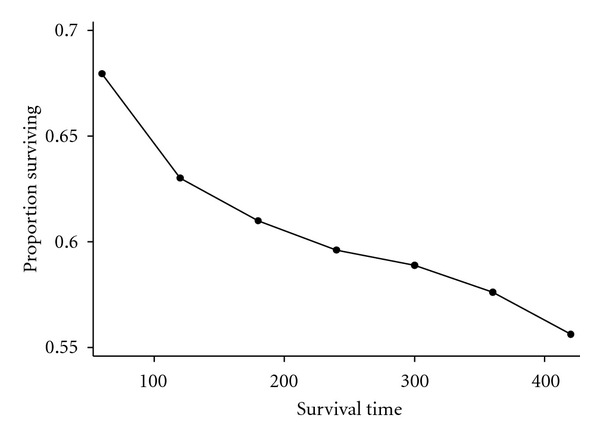
Overall proportion of pediatric patients with leukemia surviving by 5-year interval.

**Figure 4:**
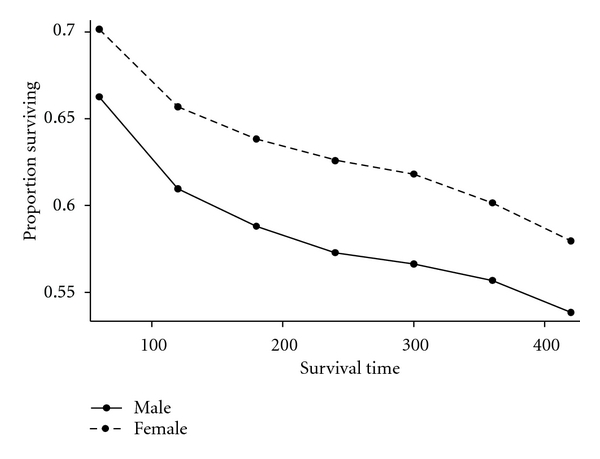
Proportion of pediatric patients with leukemia by sex surviving, 5-year interval.

Figure 1 illustrates the overall survival curve for pediatric leukemia patients. The survival curve is demonstrated by sex with boys showing poorer survival, log rank, *P* < 0.0001 (Figure 2).  Figure 3 shows the 5-year interval overall survival of pediatric patients with leukemia, while Figure 4 demonstrates the similar time interval for survival by sex.

## 4. Discussion

Survival disparity by the sex of the pediatric patient with leukemia has been observed since the nineteen sixties; however, what remains to be fully grasped are the factors responsible for this persisting survival difference between boys and girls. Girls continue to demonstrate survival advantage relative to boys. We conducted a survival assessment, analyzing data spanning a 34-year period (1973–2006) to examine whether or not sex distinction in pediatric leukemia survival still exists, as well as whether we can explain survival variability by sex.

Our assessment has several relevant findings. First, we have validated the observed sex disparity in pediatric leukemia survival using a large and long-term dataset. Second, boys are more likely to show poorer outcome of survival compared to girls. Third, the crude survival in this cohort is associated with race, age at diagnosis, year of diagnosis, number of primaries, and tumor cell type. Finally, survival differences by sex are not explained by the differences in radiation therapy received, age at tumor diagnosis, year of diagnosis, race, or tumor cell type.

Studies over the past years [5–17] have repeatedly shown that after diagnosis of pediatric leukemia, boys present with poorer survival. In this study, we wanted to see whether using a large sample and long-term data will help explain the ongoing variance in leukemia survival comparing boys to girls. In doing this, we considered factors within the dataset that might possibly influence survival in this cohort. After having adjusted for these factors, we have shown that boys are more likely to die from leukemia. This finding is supported by previous studies [2–4, 8–17].

Pediatric leukemia survival may be associated with the tumor cell type at diagnosis [22]. The patients with T-cell leukemia have poorer survival, and in our study, boys were more likely to have T-cell leukemia. Our finding supports previous studies on sex differences in leukemia prognosis [22, 23]. The leukemia cell type remains in part an alternative explanation to the observed disparity in survival by sex, since most of the patients who had T-cell type were boys, and survival was poorer among boys in our study. One would think, after controlling for these factors, the observed variance would not persist, but that was not the case in this dataset. Therefore, there appears to be a biological explanation beyond the cell type for sex disparity in pediatric leukemia survival. It is plausible to suspect XY chromosomal instability as a possible contribution to abnormal cellular proliferation, thus resulting in a biologically aggressive leukemia among male pediatric patients. Also, it might be possible that testosterone or estrogen may play a small role in pediatric leukemia. However, in our data, these hormones used as proxy by categorizing age at diagnosis into two groups (0–9 versus 10–19 years) and examining survival by sex while adjusting for the effect of these hormones did not alter the effect of sex on survival. However, our data showed in the univariable Cox regression model that children aged 10–19 years were two times as likely to experience mortality compared to children aged 0–9 years. Nevertheless, it is not clear if this observation is explained by the effect of hormones on tumor prognosis, and if this is the case, which hormone, estrogen, or testosterone might be causing this effect. Since tumor cell type significantly influenced survival, and older children were more likely to be diagnosed with T-cell type which is associated with poor survival, this may explain in part why survival was poorer for children aged 10–19 years at the time of tumor diagnosis. The Mantel-Haenszel stratification analysis performed to examine the sex difference on the effect of dichotomous age group at diagnosis showed poorer survival for boys in the age group of 10–19 years relative to girls in the same age group.

Racial/ethnic variation in pediatric leukemia survival has been shown by previous findings [24–26]. Our results support previous studies, since we found race to be an independent predictor of survival in pediatric leukemia. The black children with leukemia presented with higher mortality relative to their white counterparts. Indeed, our study found that black children were 54% more likely to die from leukemia compared to white children. Race is a difficult concept to explain. Information on race and the factors defining it remains unclear. Whereas we have observed racial variation in pediatric leukemia survival, it is not very clear what this translates to in terms of cancer survival. Therefore, the racial variation observed in this dataset may be related to racial disparities in socioeconomic factors, life-style variables, or biology, factors which were not available in the dataset used for this study.

We have also shown that radiation as a monotherapy did not improve survival of children diagnosed with leukemia and treated for the disease. This finding supports the limitation of monotherapy in pediatric cancer therapeutics. However, our study also showed that in combination with other therapies, radiation therapy provides a survival advantage. This is verified based on findings using the multivariable model, which allows us to simultaneously adjust for the effect of other covariates, while examining the effect of radiation therapy on survival of these children.

Survival varied significantly by the age at diagnosis. Children less than one year of age had the poorest survival, which may be explained by their immature immune system. The immune responsiveness to tumor antigen has been well documented [27]. Children diagnosed with leukemia who are less than six months of age are less likely to mount immune response to the tumor-specific antigen, and therefore, the immunologic surveillance of malignancy is significantly compromised at this age group [28]. It also appears that the older the children, the better the survival, but this was not a linear trend since the best survival was seen among children aged 1–4 years.

Children continue to be diagnosed with more than one primary tumor. For example, children with retinoblastoma have an increased risk of developing leukemia [29]. Children with more than one primary tumor were shown in our study to have poorer survival. It is plausible that survival was poorer among children with more than one primary. This might be due in part to the interaction in treatment (radiation, chemotherapy) that may further lower the immune responsiveness to the tumor, and thereby decrease survival among children with more than one primary. However, drug interaction in pediatric cancer is not very well understood, given the present stage of our knowledge in pediatric cancer therapeutics, and the potentials for adverse survival remains.

The year of diagnosis has been shown in our study to significantly influence leukemia survival. We examined this variable using 5-year intervals and found that children diagnosed during the early years of SEER registry (1973−1997) had poorer survival relative to those diagnosed in later years of registry. Indeed, we observed a trend in the 5-year of diagnosis groupings. Because the year of diagnosis influences the survival due to the changes in the treatment protocol over time, and the observation that the year of diagnosis is a time-dependent variable, we stratified the analysis by the year of diagnosis and found significant variation in survival by sex. This observation may very well be explained by the birth cohort effect or improvement in the treatment protocol. We could have assessed the birth cohort and the treatment effect as possible explanations for the trend in survival by year of diagnosis; however, the SEER data set is limited with respect to some treatments, namely, chemotherapy, as well as the lack of information regarding known effective treatments such as surgery.

The nature of entry into a cohort in a survival study is very important in the examination of the survival time (median, mean, and percentile). The SEER dataset used in this survival study obtained information from newly diagnosed pediatric patients with leukemia, some who entered the study at the beginning of the dataset creation (1973), and some who entered the study at the time where the most recent data on pediatric leukemia is available at the National Cancer Institute (2006). Likewise, data in this study included those who were diagnosed and died before the beginning of the survival time in this study. With this variability, it is difficult to estimate the precise median survival time for the overall cohort as well as the sex cohort. We estimated the survival percentage in this study based on our understanding that the proportion of those who died at the entry differs from those who died towards the end of the study due precisely to the variation in the length of followup. Whereas our description of survival time in this study allows one to understand the effect of sex and other covariates on survival, this description should be interpreted with caution due to this significant variation in the length of followup in the SEER dataset. In addition, due to this variability, we performed stratified analysis by the year of diagnosis in order to remove this imbalance and to present a valid hazard of dying, given the effect of sex.

Despite the strength of this study, given its potential to clearly determine the effect of sex on pediatric leukemia survival, there are a few limitations. First, our analyses are based on a retrospective design inspite of a well-organized, valid, and reliable SEER dataset. However, such dataset has a tendency for missing information on important variables. For example, we were not able to have substantial information on the radiation status of most of the patients. In addition, there was no information on chemotherapy received for leukemia. Secondly, because we used preexisting data, we were not able to assess or address confounding variables known to influence pediatric leukemia survival. Therefore, this study may be influenced by unmeasured and unknown confounding. And despite controlling for age at diagnosis, number of primaries, year of diagnosis, radiation, and race, it is possible that residual confounding might have influenced our results. However, it is highly unlikely that our findings of survival variability by sex in pediatric leukemia are driven solely by these confounding (unmeasured and residual), since no matter how sophisticated a model used to control for confounding, residual confounding is likely to influence the result of epidemiologic study [23].

## 5. Conclusions

In summary, this study showed a survival difference by sex of children with leukemia after controlling for the effect of race, age at diagnosis, year of diagnosis, number of primaries, and tumor cell type, which were the factors that influenced survival. Therefore, given the persisting sex differences in pediatric leukemia survival in this large dataset and long-term assessment, there is need to further examine biological variability in leukemia including sex variance in response to treatment.

## Figures and Tables

**Table:** (a)

Covariates	Boys	Girls	*χ* ^2^ (df)	*P* value
	*n* (%)	*n* (%)		
Race			3.81 (3)	0.28
White	7087 (88.2)	5348 (81.1)		
Black	657 (7.6)	555 (8.4)		
Others	843 (9.8)	663 (10.1)		
Unknown	35 (0.4)	27 (0.4)		
Age at diagnosis (yrs)			49.76 (4)	<0.0001
<1.0	386 (4.5)	385 (5.8)		
1.0−4.0	3411 (39.6)	2650 (40.2)		
5.0–9.0	1871 (21.7)	1550 (23.5)		
10.0–14.0	1450 (16.8)	1094 (16.5)		
15.0−19.0	1504 (17.4)	914 (13.9)		
Year of diagnosis			5.72 (6)	0.46
1973–1977	683 (7.9)	488 (7.4)		
1978–1982	641 (7.4)	516 (7.8)		
1983–1987	711 (8.2)	550 (8.3)		
1988–1992	865 (10.0)	661 (10.0)		
1993–1998	1290 (15.0)	1005 (15.2)		
1999–2002	2167 (25.1)	1720 (26.1)		
2003–2006	2265 (26.3)	1653 (25.1)		
Number of primaries			3.16 (1)	0.07
1.0	8499 (98.6)	6475 (98.2)		
≥ 2.0	123 (1.4)	118 (1.8)		
Survival time (months)				<0.0001
Median	45.0	51.0		
Mean (sd)	79.32 (88.4)	85.87 (93.06)		
Mortality status			23.6 (1)	<0.0001
Alive	5815 (67.4)	4689 (71.1)		
Dead	2807 (32.6)	1904 (28.9)		
Radiation			23.2 (1)	<0.0001
Yes	1622 (18.8)	1043 (15.8)		
No	7000 (81.2)	5550 (84.2)		

Abbreviations and notes: sd = standard deviation. The significance level is 0.01 (1% type 1 error tolerance).

**Table:** (b)

		Survival percentage by sex	
Survival time interval (months)	Overall survival (%)	Boys (%)	Girls (%)
0–60	67.9	66.3	70.1
60–120	63.0	60.9	65.7
120–180	61.0	58.8	63.8
180–240	59.6	57.3	62.6
240–300	58.9	56.6	61.8
300–360	57.1	55.7	60.1
360–420	55.6	53.9	58.0

Notes: the maximum followup time was 408 months. The survival interval for the last row is 360–408 months but presented as 360–420 to meet the five-year survival cutoff point.

**Table 2:** Mortality associated with sex and other factors in pediatric patients diagnosed with leukemia, 1973–2006.

Covariates	Hazard ratio (HR)	99% CI	*P*
Sex			
Male	1.00	Ref	Ref
Female	0.86	0.80–0.93	<0.0001
Race			
White	1.00	Ref	Ref
Black	1.54	1.36–1.74	<0.0001
Age at diagnosis (yrs)			
<1.0	1.00	Ref	Ref
1.0−4.0	0.26	0.22–0.30	<0.0001
5.0–9.0	0.33	0.28–0.39	<0.0001
10.0–14.0	0.56	0.48–0.66	<0.0001
15.0 −19.0	0.89	0.77–1.04	0.05
Number of primaries			
1.0	1.00	Ref	Ref
≥ 2.0	1.75	1.40–2.20	<0.0001
Beam radiation			
Yes	1.00	Ref	Ref
No	0.84	0.77–0.92	<0.0001

Notes and abbreviations: the significance level was 0.001. CI = confidence interval, ref = reference group or class.

**Table 3:** Mortality associated with sex in pediatric patients diagnosed with leukemia, 1973–2006.

Covariates	AHR	99% CI	*P*
Sex			
Boys	1.0	Ref	Ref
Girls	0.88	0.81–0.95	<0.0001
Race			
White	1.0	Ref	Ref
Black	1.47	1.31–1.66	<0.0001
Age at diagnosis (years)			
<1.0	1.0	Ref	Ref
1.0−4.0	0.24	0.21–0.28	<0.0001
5.0–9.0	0.31	0.26–0.37	<0.0001
10.0–14.0	0.54	0.46–0.62	<0.0001
15.0−19.0	0.83	0.71–0.96	0.001
Year of diagnosis			
1973–1977	1.0	Ref	Ref
1978–1982	0.70	0.61–0.81	<0.0001
1983–1987	0.57	0.49–0.65	<0.0001
1988–1992	0.40	0.35–0.47	<0.0001
1993–1997	0.36	0.32–0.42	<0.0001
1998–2002	0.30	0.26–0.34	<0.0001
2003–2006	0.27	0.23–0.31	<0.0001
Number of primaries			
1.0	1.0	Ref	Ref
≥ 2.0	1.32	1.10–1.33	0.002
Radiation			
Yes	1.0	Ref	Ref
No	1.21	1.10–1.33	<0.0001

Notes and abbreviations: the significance level was 0.01. CI = confidence interval. AHR = adjusted hazard ratio, ref = the reference group or class.
